# Clinical diagnostic whole genome sequencing in a paediatric population: experience from our WGS genetics clinic

**DOI:** 10.1186/1753-6561-6-S6-O11

**Published:** 2012-10-01

**Authors:** Elizabeth Worthey, David Bick, David Dimmock, Mary Shimoyama, Regan Veith, George Kowalski, Bradley Taylor, Brandon Wilk, Sharon Tsaih, Jeremy Harris, Michael Tschannen, Jaime Wendt-Andrae, Ken Brodie, Howard Jacob

**Affiliations:** 1 Human and Molecular Genetics Center, The Medical College of Wisconsin, Milwaukee 53226, WI, USA; 2 The Department of Pediatrics, The Medical College of Wisconsin, Milwaukee 53226, WI, USA; 3 The Children's Hospital of Wisconsin, 9000 W. Wisconsin Avenue, Milwaukee, 53226, WI, USA; 4 The Department of Surgery, The Medical College of Wisconsin, Milwaukee 53226, WI, USA; 5 The Department of Physiology, The Medical College of Wisconsin, Milwaukee 53226, WI, USA

## Background

We are in an unprecedented era where the genome of an individual can be sequenced in a day for low cost in a single sequencing run. The availability this information is changing and challenging the practice of medicine. At The Medical College of Wisconsin (MCW) we have been running a whole genome sequencing (WGS) based genetics clinic for over a year. WGS together with the processes and tools developed for analysis and interpretation are being used to identify disease-causing mutations in rare pediatric disorders in children being treated at our affiliated Children's Hospital of Wisconsin.

## Materials and methods

We have developed a fully integrated WGS process for clinical diagnosis: from sequence generation through to interpretation and reporting, including follow-up and counselling tailored specifically to WGS testing. Our initial pilot program has been extended to a Children's Hospital of Wisconsin clinic providing dedicated genetics staff. This required development of a series of CAP/ CLIA compliant protocols and pipelines for dealing with samples and returning reports, and has spurred ongoing development of tools including a platform for electronic health record (EHR) data extraction. Clinically validated WGS analysis tools were also developed to support determination of whether the variant is: (1) a likely error, (2) known to be causally linked with human disease, (3) of a type likely to lead to altered function thereby giving rise to a phenotypic change, and/or (4) within a gene with a function associated or relevant to the patient phenotype (including identification of causative disease genes). They support consideration of variant and functional information simultaneously, as required for interpretation and reporting. Development of this clinic also required formation of institutional review bodies, definition of a structure for counselling, and development of criteria for appropriate reporting and return of WGS-based findings.

## Results

Of 22 cases approved for clinical WGS in the MCW pilot program, 15 have been analyzed and/or reported. Our protocols and procedures have been requested and shared with other sites moving into this area. Our tools have been clinically validated and are in use, and continue to be developed to add more sophisticated algorithms to support interpretation. Use of these tools has rendered diagnoses in some but not all cases and in some cases; these diagnoses have led to alteration in treatment in some but not all cases [1,2]. In addition these tools are being applied to uncovering causative mutations for dozens of additional patients as part of various research project collaborations.

## Conclusions

WGS and subsequent analysis can be used to identify causative mutations and result in diagnosis in patients with rare disorders today. Reaching this stage requires expert use of sophisticated bioinformatics tools for interpretation. The diagnosis can be used to alter treatment in some cases. We will: (1) provide examples where WGS analysis has rendered a molecular diagnosis in individual patients or families in the setting of novel disease, (2) highlight challenges faced, and (3) focus on the nature of the tools developed and how they are used in a clinical setting.
